# Supplementary material for: Enterovirus 71 seroepidemiology in Taiwan in 2017 and comparison of those rates in 1997, 1999 and 2007
Source: PLoS One. 2019 Oct 17;14(10):e0224110. doi: 10.1371/journal.pone.0224110 (PMC6797108; doi:10.1371/journal.pone.0224110)
Supplement: S1 File — (PDF) [file pone.0224110.s001.pdf]

台灣城鄉之社區腸病毒 71 型血清流行病學調查-幼兒問卷

EV71 Seroepidemiology study in urban and rural areas in Taiwan:

Questionnaire for preschool children

Date：___/___/___

一、基本資料與兒童照護

First, basic information and child care

1.被抽血人姓名：<br>1. Name of the study subject	性別：<br>gender:	生日：民國 年 月 日<br>Date of birth： / /
2.家長姓名：<br>2.Parent's name:<br><br>填表人為：<br>The name who fills in the form:	聯絡方式(手機)<br>Contact information (mobile phone)	(電話)<br>(phone)
3.地址<br>3. Address		
4.這個住處已經住了____年<br>4. This place has lived for (how many)years	住家是在：（請勾選）<br>□城市區, □鄉村區, □城市邊緣地(市郊) ,□工業區<br>The home is at: (please tick)<br>□ urban area, □ rural area, □ urban fringe (suburban), □ industrial area	
5.同住家庭人數共:_____人<br>5. Living with family members:_______ (how many) People		
6.小朋友手足數:(_________人)<br>為______兄，年齡分別為：(________________)<br>為______姊，年齡分別為：(________________)<br>為______弟，年齡分別為：(________________)<br>為______妹，年齡分別為：(________________)<br>6.Number of children, brothers and sisters: (_________ people)<br>______(how many) elder brothers, the ages are: (________________)<br>______(how many) elder sister, the ages are: (________________)<br>______(how many) younger brothers, the ages are: (________________)<br>______(how many) younger sisters, the ages are: (________________)		
7.小朋友是否有慢性疾病<br>7. Does the child have chronic diseases?	□有,（請註明：______________________ ）<br>□無，<br>□ Yes, (please specify:) □No	
8.小朋友是否被餵過母奶<br>8. Has the child been fed breast milk?	□是:從___ 月大到____ 月大,<br>□否<br>□ Yes: From month to month,<br>□No	
9.小朋友是上幼稚園或托兒所?<br>9. Is the child attending a kindergarten or nursery?	□是<br>□否<br>□Yes<br>□No	
10.小朋友若未上幼稚園或托兒所,主要照顧者為：□褓姆,□母親,□父親,□祖父母,□其他：___________<br>10.If the child is not attending a kindergarten or nursery school, the main caregivers are:<br>□Babysitter, □ mother, □father, □ grandparents, □ others: __________		

11.家中是否有人為B型肝炎帶原者:<br>□有(請註明: ____)<br>□無<br>11. Is there anyone in the family who is a hepatitis B carrier:<br>□ Yes (please specify: ____)<br>□No
12.家中主要飲水來源：□自來水,□井水, □泉水,□其他：_______________________<br>12. Main sources of drinking water in the home: □ tap water, □ well water, □ spring water, □ other: ____________

二、腸病毒相關疾病及接觸史

Second, enterovirus-related diseases and exposure history

題目<br>topic	請選擇符合您的選項<br>Please select the option that matches your choice
1.小孩是否得過手足口症？<br>1. Does the child have a medical history of hand, foot and mouth disease?	□有，幾次：□1, □2, □3, □4, □大於 5 次<br>□無<br>□不知<br>□ Yes, several times: □1, □2, □3, □4, □ more than 5 times<br>□No<br>□ I don't know
2.小孩是否得過咽峽炎？<br>2. Does the child have a medical history of herpangina?	□有，幾次：□1, □2, □3, □4, □大於 5 次<br>□無<br>□不知<br>□ Yes, several times: □1, □2, □3, □4, □ more than 5 times<br>□No<br>□ I don't know
3.家人中是否有人得過咽峽炎或手足口症？<br>3. Did anyone in the family have herpangina or hand, foot and mouth disease in the past?	□有(與小孩的關係為：______),<br>□無<br>□不知<br>□ Yes (the relationship with children is:),<br>□No<br>□ I don't know
4.小孩的同學是否得過咽峽炎或手足口症？<br>4. Did the child's classmates have herpangina or hand, foot and mouth disease in the past?	□有<br>□無<br>□不知<br>□ Yes<br>□No<br>□ I don't know
5.小孩是否曾與手足口症或咽峽炎患者接觸？<br>5. Did the child have contact with patients with hand, foot and mouth disease or herpangina?	□有，請勾選以下<br>接觸方式：____握手，____摟抱，____親吻，____餵食，____遊玩，<br>__其他方式：註明_________________________________<br>□無<br>□不知<br>□ Yes, please tick the following<br>Contact:<br>□shaking hands,<br>□hugging,<br>□kissing,

	□sharing food,<br>□playing,<br>Other ways: please specify ______________________________<br>□No<br>□ I don't know

三、疫苗接種史 (請問您打了下列疫苗否？請按健康手冊之疫苗接種紀錄填)：

Third, the history of vaccination (Do you have the following vaccine? Please fill in according to the vaccination record of the children health handbook):

健康手冊之疫苗接種紀錄可否影印給我們存檔確認以下資料□可　　□否

Can the vaccination record of the children health handbook be photocopied to us to confirm the following information?□Yes □ No

疫苗名稱<br>Vaccine name	★「有」施打（以下請勾）<br>★ "Yes" Vaccination (Please check below)	若「有」施打，共幾劑<br>If you have a vaccine, how many doses were received	若「有」施打，最後一劑日期<br>If you have a vaccine, please write down the date of last dose	★「無」施打（以下請勾）<br>★"None" vaccine (Please check below)	★「不知是否有」施打（以下請勾）<br>★ "I don't know Whether the vaccine was received
1.B 型肝炎疫苗：<br>Hepatitis B vaccine	□有<br>□Yes	幾劑<br>how many ____ doses	年　月　日<br>___year___month ___day	□無<br>□NO	□不知<br>□Unknown
2.卡介苗核 BCG 疫苗<br>(Bacillus Calmette-Guérin (BCG) vaccine	□有<br>□Yes	幾劑<br>how many ____ doses	年　月　日<br>___year___month ___day	□無<br>□NO	□不知<br>□Unknown
3.五合一疫苗<br>DTaP-Hib-IPV<br><br>Diphtheria and tetanus toxoid with acellular pertussis vaccine, Haemophilus influenzae type b vaccine, Inactivated polio vaccine	□有<br>□Yes	幾劑<br>how many ____ doses	年　月　日<br>___year___month ___day	□無<br>□NO	□不知<br>□Unknown
4.肺炎鏈球菌疫苗<br>Pneumococcal conjugate vaccine	□有<br>□Yes	幾劑<br>how many ____ doses	年　月　日<br>___year___month ___day	□無<br>□NO	□不知<br>□Unknown

5.痲疹/腮腺炎/德國痲疹疫苗<br>Measles, mumps, and rubella vaccine	□有<br>□Yes	幾劑<br>how many ____ doses	年 月 日<br>___year___month ___day	□無<br>□NO	□不知<br>□Unknown
6.水痘疫苗<br>Chickenpox Vaccine	□有<br>□Yes	幾劑<br>how many ____ doses	年 月 日<br>___year___month ___day	□無<br>□NO	□不知<br>□Unknown
7.日本腦炎疫苗<br>Japanese encephalitis vaccine	□有<br>□Yes	幾劑<br>how many ____ doses	年 月 日<br>___year___month ___day	□無<br>□NO	□不知<br>□Unknown
8.A 型肝炎疫苗<br>Hepatitis A vaccine	□有<br>□Yes	幾劑<br>how many ____ doses	年 月 日<br>___year___month ___day	□無<br>□NO	□不知<br>□Unknown
