# Supplementary material for: Enterovirus 71 seroepidemiology in Taiwan in 2017 and comparison of those rates in 1997, 1999 and 2007
Source: PLoS One. 2019 Oct 17;14(10):e0224110. doi: 10.1371/journal.pone.0224110 (PMC6797108; doi:10.1371/journal.pone.0224110)
Supplement: S2 File — (PDF) [file pone.0224110.s002.pdf]

台灣城鄉之社區腸病毒 71 型血清流行病學調查-學生幼兒問卷

EV71 Seroepidemiology study in urban and rural areas in Taiwan: Questionnaire for students

Date：____/____/

一、基本資料

First, basic information

<table>
<tr><td colspan="2">1.被抽血人姓名：
1. Name of the study subject</td><td>性別：
gender:</td><td colspan="2">生日：民國　年　月　日
Date of birth：____/____/</td></tr>
<tr><td colspan="2">2.家長姓名：
1. Name of the person being drawn
填表人為：
The name who fills in the form:</td><td colspan="2">聯絡方式(手機)
Contact information (mobile phone)</td><td>(電話)
(phone)</td></tr>
<tr><td colspan="5">3.地址
3. Address</td></tr>
<tr><td>4.這個住處已經住了____年
4. This place has lived for (how many) years</td><td colspan="4">住家是在：（請勾選）
□城市區, □鄉村區, □城市邊緣地(市郊) ,□工業區
The home is at: (please tick)
□ urban area, □ rural area, □ urban fringe (suburban), □ industrial area</td></tr>
<tr><td colspan="5">5.同住家庭人數共:_____人
5. Living with family members:_______ (how many) People</td></tr>
<tr><td colspan="5">6.小朋友手足數:(_________人)
為______兄，年齡分別為：（_________________)
為______姊，年齡分別為：（_________________)
為______弟，年齡分別為：（_________________)
為______妹，年齡分別為：（_________________)
6.Number of children, brothers and sisters: (_________ people)
______(how many) elder brothers, the ages are: (_________________)
______(how many) elder sister, the ages are: (_________________)
______(how many) younger brothers, the ages are: (_________________)
______(how many) younger sisters, the ages are: (_________________)</td></tr>
<tr><td>7.小朋友是否有慢性疾病
7. Does the child have chronic diseases?</td><td colspan="4">□有,（請註明：______________________ ）
□無，
□ Yes, (please specify:) □No</td></tr>
<tr><td>8.小朋友是否被餵過母奶
8.Has the child been fed breast milk?</td><td colspan="4">□是:從___ 月大到____ 月大,
□否
□Yes
□No</td></tr>
<tr><td colspan="5">9.家中是否有人為B型肝炎帶原者:
□有(請註明: ____)
□無
9. Is there anyone in the family who is a hepatitis B carrier:
□ Yes (please specify: __________________)
□No</td></tr>
<tr><td colspan="5">10.家中主要飲水來源：□自來水,□井水,□泉水,□其他：_________________________
10. Main sources of drinking water in the home: □ tap water, □ well water, □ spring water, □ other: _____________</td></tr>
</table>

二、腸病毒相關疾病及接觸史

Second, enterovirus-related diseases and exposure history

題目<br>topic	請選擇符合您的選項<br>Please select the option that matches your choice
1.小孩是否得過手足口症？<br>1. Does the child have a medical history of hand, foot and mouth disease?	□有，幾次：□1, □2, □3, □4, □大於 5 次<br>□無<br>□不知<br>□ Yes, several times: □1, □2, □3, □4, □ more than 5 times<br>□No<br>□ I don’t know
2.小孩是否得過咽峽炎？<br>2. Does the child have a medical history of herpangina?	□有，幾次：□1, □2, □3, □4, □大於 5 次<br>□無<br>□不知<br>□Yes, several times: □1, □2, □3, □4, □ more than 5 times<br>□No<br>□ I don’t know
3.家人中是否有人得過咽峽炎或手足口症？<br>3. Did anyone in the family have herpangina or hand, foot and mouth disease in the past?	□有(與小孩的關係為：______),<br>□無<br>□不知<br>□Yes (the relationship with children is: --------),<br>□No<br>□ I don’t know
4.您的鄰居是否得過咽峽炎或手足口症？<br>4.Did your neighbors have herpangina or hand, foot and mouth disease in the past?	□有<br>□無<br>□不知<br>□Yes<br>□No<br>□I don’t know
5.小孩的同學是否得過咽峽炎或手足口症？<br>5.Did the child's classmates have had herpangina or hand, foot and mouth disease in the past?	□有<br>□無<br>□不知<br>□ Yes<br>□No<br>□ I don’t know
6.小孩是否曾與手足口症或咽峽炎患者接觸？<br>6. Did the child have contact with patients with hand, foot and mouth disease or herpangina?	□有，請勾選以下<br>接觸方式：____握手，____摟抱，____親吻，____餵食，____遊玩，____其他方式：註明______________________________<br>□無<br>□不知<br>□ Yes, please tick the following<br>Contact:<br>□shaking hands,<br>□hugging,<br>□kissing,<br>□sharing food,<br>□playing,<br>Other ways: please specify ______________________________

	□No<br>□ I don't know

三、疫苗接種史 (請問您打了下列疫苗否？請按健康手冊之疫苗接種紀錄填)：

Third, the history of vaccination (Do you have the following vaccine? Please fill in according to the vaccination record of the children health handbook):

健康手冊之疫苗接種紀錄可否影印給我們存檔確認以下資料□可　□否

Can the vaccination record of the children health handbook be photocopied to us to confirm the following information?□Yes □ No

疫苗名稱<br>Vaccine name	★「有」施打（以下請勾）<br>★ "Yes" Vaccination (Please check below)	若「有」施打，共幾劑<br>If you have a vaccine, how many doses were received	若「有」施打，最後一劑日期<br>If you have a vaccine, please write down the date of last dose	★「無」施打（以下請勾）<br>★"None" vaccine (Please check below)	★「不知是否有」施打（以下請勾）<br>★ "I don't know Whether the vaccine was received
1.B 型肝炎疫苗：<br>Hepatitis B vaccine	□有<br>□Yes	幾劑<br>how many ____ doses	年　月　日<br>___year___month ___day	□無<br>□NO	□不知<br>□Unknown
2.卡介苗核 BCG 疫苗<br>Bacillus Calmette-Guérin (BCG) vaccine	□有<br>□Yes	幾劑<br>how many ____ doses	年　月　日<br>___year___month ___day	□無<br>□NO	□不知<br>□Unknown
3.五合一疫苗<br>DTaP-Hib-IPV Diphtheria and tetanus toxoid with acellular pertussis vaccine, Haemophilus influenzae type b vaccine, Inactivated polio vaccine	□有<br>□Yes	幾劑<br>how many ____ doses	年　月　日<br>___year___month ___day	□無<br>□NO	□不知<br>□Unknown
4.肺炎鏈球菌疫苗<br>Pneumococcal conjugate vaccine	□有<br>□Yes	幾劑<br>how many ____ doses	年　月　日<br>___year___month ___day	□無<br>□NO	□不知<br>□Unknown
5.麻疹/腮腺炎/德國麻疹疫苗<br>Measles, mumps, and rubella vaccine	□有<br>□Yes	幾劑<br>how many ____ doses	年　月　日<br>___year___month ___day	□無<br>□NO	□不知<br>□Unknown
6.水痘疫苗<br>Chickenpox	□有	幾劑	年　月　日	□無	□不知

Vaccine	□Yes	how many _____ doses	___year___month ___day	□NO	□Unknown
7.日本腦炎疫苗<br>Japanese encephali-tis vaccine	□有<br>□Yes	幾劑<br>how many _____ doses	年 月 日<br>___year___month ___day	□無<br>□NO	□不知<br>□Unknown
8.A 型肝炎疫苗<br>Hepatitis A vaccine	□有<br>□Yes	幾劑<br>how many _____ doses	年 月 日<br>___year___month ___day	□無<br>□NO	□不知<br>□Unknown
