# Supplementary material for: Enterovirus 71 seroepidemiology in Taiwan in 2017 and comparison of those rates in 1997, 1999 and 2007
Source: PLoS One. 2019 Oct 17;14(10):e0224110. doi: 10.1371/journal.pone.0224110 (PMC6797108; doi:10.1371/journal.pone.0224110)
Supplement: S3 File — (PDF) [file pone.0224110.s003.pdf]

台灣城鄉之社區腸病毒 71 型血清流行病學調查-成人

EV71 Seroepidemiology study in urban and rural areas in Taiwan:

Questionnaire for adults

Date：____/____/

一、基本資料

First, basic information

1.被抽血人姓名：<br>1. Name of the study subject	性別：<br>gender:	生日：民國　年　月　日<br>Date of birth：___/___/
聯絡方式(手機)<br>Contact information (mobile phone)	(電話)<br>(phone)	
2.地址<br>2. Address		
3.這個住處已經住了____年<br>3.This place has lived for (how many)years	住家是在：（請勾選）<br>☐城市區, ☐鄉村區, ☐城市邊緣地(市郊）,☐工業區<br>The home is at: (please tick)<br>☐ urban area, ☐ rural area, ☐ urban fringe (suburban), ☐ industrial area	
4.同住家庭人數共:_____人<br>4.Living with family members:_______ (how many) People		
5.您是否有小孩，有，請填以下：<br>為_____男，年齡分別為：(_______________)<br>為_____女，年齡分別為：(_______________)<br>5. Do you have children, yes, please fill in the following:<br>_____(how many) sons, the ages are: (_______________)<br>_____(how many) daughters, the ages are: (_____________)		
6.您是否有慢性疾病?<br>6. Do you have a chronic disease?	☐有,（請註明：____ ）<br>☐無，<br>☐ Yes, (please specify:) ☐No	
7.您是否為B型肝炎帶原者?<br>7.Are you a hepatitis B carrier:<br>☐是,<br>☐否,<br>☐不知<br>☐ Yes<br>☐No<br>☐ I don’t know		
8.家中是否有人為B型肝炎帶原者:<br>8. Is there anyone in the family who is a hepatitis B carrier:<br>☐有(請註明: ____)<br>☐無<br>☐ Yes (please specify:______________________________________)<br>☐No		
9.家中主要飲水來源：☐自來水,☐井水, ☐泉水,☐其他：______________________		

9. Main sources of drinking water in the home: □ tap water, □ well water, □ spring water, □ other: ____________

二、腸病毒相關疾病及接觸史

Second, enterovirus-related diseases and exposure history

題目	請選擇符合您的選項
1.您是否得過手足口症？<br>1.Did you have hand, foot and mouth disease in the past?	□有，幾次：□1, □2, □3, □4, □大於 5 次<br>□無<br>□不知<br>□ Yes, several times: □1, □2, □3, □4, □ more than 5 times<br>□No<br>□ I don' t know
2.您是否得過咽峽炎？<br>2.Did you have herpangina in the past?	□有，幾次：□1, □2, □3, □4, □大於 5 次<br>□無<br>□不知<br>□ Yes, several times: □1, □2, □3, □4, □ more than 5 times<br>□No<br>□ I don' t know
3.家人中是否有人得過咽峽炎或手足口症？<br>3.Did anyone in the family have herpangina or hand, foot and mouth disease in the past?	□有(與您的關係為：______),<br>□無<br>□不知<br>□ Yes (the relationship with you is:)<br>□No<br>□ I don' t know
4.您的鄰居是否得過咽峽炎或手足口症？<br>4.Did your neighbors have herpangina or hand, foot and mouth disease in the past?	□有<br>□無<br>□不知<br>□ Yes<br>□No<br>□ I don' t know
5.您是否曾與手足口症或咽峽炎患者接觸？<br>5.Did you have contact with patients with hand, foot and mouth disease or herpangina?	□有，請勾選以下<br>接觸方式：____握手，____摟抱，____親吻，____餵食，____遊玩，__其他方式：註明______________________________<br>□無<br>□不知<br>□ Yes, please tick the following<br>Contact:<br>□shaking hands,<br>□hugging,<br>□kissing,<br>□sharing food,<br>□playing,<br>Other ways: please specify ______________________________<br>□No<br>□ I don' t know
6.您的小孩是否得過手足口症？	□有，幾次：□1, □2, □3, □4, □大於 5 次

6. Did your children have hand, foot and mouth disease in the past?	☐無<br>☐不知<br>☐ Yes, several times: ☐1, ☐2, ☐3, ☐4, ☐ more than 5 times<br>☐No<br>☐ I don' t know
7.您的小孩是否得過咽峽炎？<br>7. Did your children have herpangina in the past?	☐有，幾次：☐1, ☐2, ☐3, ☐4, ☐大於5次<br>☐無<br>☐不知<br>☐Yes, several times: ☐1, ☐2, ☐3, ☐4, ☐ more than 5 times<br>☐No<br>☐ I don' t know

三、疫苗接種史

Third, the history of vaccination

請問您打了下列疫苗否？

Do you have the following vaccine

疫苗名稱<br>Vaccine name	★「有」施打（以下請勾）<br>★ "Yes" Vaccination (Please check below)	若「有」施打，共幾劑<br>If you have a vaccine, how many doses were received	若「有」施打，最後一劑日期<br>If you have a vaccine, please write down the date of last dose	★「無」施打（以下請勾）<br>★"None" vaccine (Please check below)	★「不知是否有」施打（以下請勾）<br>★ "I don't know Whether the vaccine was received
1. A型肝炎疫苗<br>Hepatitis A vaccine	☐有<br>☐Yes	幾劑<br>how many ____ doses	年 月 日<br>___year___month ___day	☐無<br>☐NO	☐不知<br>☐Unknown
2. B型肝炎疫苗：<br>Hepatitis B vaccine	☐有<br>☐Yes	幾劑<br>how many ____ doses	年 月 日<br>___year___month ___day	☐無<br>☐NO	☐不知<br>☐Unknown
3.水痘疫苗<br>Chickenpox Vaccine	☐有<br>☐Yes	幾劑<br>how many ____ doses	年 月 日<br>___year___month ___day	☐無<br>☐NO	☐不知<br>☐Unknown
