# Supplementary material for: Enterovirus 71 seroepidemiology in Taiwan in 2017 and comparison of those rates in 1997, 1999 and 2007
Source: PLoS One. 2019 Oct 17;14(10):e0224110. doi: 10.1371/journal.pone.0224110 (PMC6797108; doi:10.1371/journal.pone.0224110)
Supplement: Attachment — Submitted filename: Response to Reviewers comment.docx [file pone.0224110.s004.docx]

Reviewers' comments:
**Comments to the Author**
Reviewer #1: The manuscript “Enterovirus 71 Seroepidemiology in Taiwan in 2017 and comparison of those rates in 1997, 1999 and 2007” provided an updated serostatus of EV71 in different region of Taiwan. This is a timely study as the threat of enterovirus infection continuously posing significant concern worldwide.
**Response:** thank you very much for your comment and appreciation.

Below are my comments for improvement.
1) Is there any patient data co-relating with the seropositive specimen? For example, successful rate of detecting viral RNAs/antigens in specimens of seropositive patients; the history of seropositive patients with enterovirus-related syndromes.
**Response:** we did not have any data of detecting viral RNAs/antigens in specimens of seropositive patients, but we did have data on enterovirus-related ( HFMD or herpangina) history of seropositive children in Table 2. For example, 34% of seropositive preschool children had history of HFMD and 24% had history of herpangina (Table 2).
2) The authors suggested that female gender could be a risk factor of EV71 seropositivity. The comparison appears to be based on the absolute number of seropositivity in between male and female. The comparison may be subjected to the biases due to disproportion of male:female ratio within the community. The authors may need to consider this point and perform normalization accordingly.
**Response:** we agree with this comment, so normalization was done for Figure 1 accordingly to minimize any disproportion of male:female ratio within the community.

3) The number of tested specimen (n) needs to be indicated in table 3.
**Response:** Thank you very much for your suggestion and we added the numbers of seropositive and tested specimen in Table 3. However, only those numbers are not available in Cambodia and we can not add them.

4) The authors suggested the lower EV71 seropositivity detected in specimen of 2007 and 2017 could be due to the absence of disease outbreak. Does the high seropositivity detected in other countries, for example, Cambodia (Table 3), correlate to an EV71 outbreak?
**Response:** We agree with your comment that the high seropositivity detected in other countries correlate to an EV71 outbreak. We mentioned it in the Discussion: Severe EV71 cases occurred more frequently in countries, such as China and Cambodia which had greater seropositive rates [7, 23, 31-33] and it indicates greater disease burden in these countries. EV71 outbreak occurred in Cambodia in 2012, it characterized by severe encephalitis with cardiovascular collapse and pulmonary edema seized international headlines and resulted in the death of at least 54 children [31], and more than 2 thousands of laboratory-confirmed EV71 cases died in China from 2008 to 2015 [7].
5) Figure 2 showed similar but delayed trend of EV71 seropositivity between specimens obtained from 1997 and 1999 to 2007 and 2017. For example, an age 1 EV71 seropositive specimen detected in 1999 will become an age 8 EV71 seropositive specimen detected in 2007. In other words, the delay could be an indication of effective infectious disease control. The authors should discuss what infectious disease control policy may have been implemented during this period of time. When comparing with data from other countries, which ways do Taiwan do better or worse?
**Response:** Thank you very much for your detailed review and suggestion. We described more infectious disease control policy in the Discussion to share our experience, which may be helpful to other countries.

Reviewer #2: Comments to the Author

The manuscript aims to explore the EV71 serostatus in Taiwan. The authors made efforts to test the EV71 neutralizing antibody with serum samples and compared EV71 serostatus among 1997, 1999, 2007 and 2017. They illustrated that the EV71 seropositive rates in children were significantly lower in 2017, compared with the rates in 1997, 1999 and 2008. These results prompt the necessity of continuous surveillance for the prevention and control of the disease infected with EV71. The main limitations of the manuscript are listed below.

1. Did the questionnaire include the subjects' vaccination history?
**Response:** The questionnaire include the subjects' vaccination history. We deposited the questionnaires as supplementary files with [Chinese-English parallel texts](https://www.linguee.com/english-chinese/translation/Chinese-English+parallel+texts.html). We did not show it in the original manuscript since EV71 vaccine is not commercially available now in Taiwan and the current vaccination did not affect EV71 serostatus. We added it in the revised manuscript since you and the readers may be curious about it.

2. As the authors indicated that the male-to-female ratio of symptomatic and/or severe enterovirus infections shifted toward males in the discussion section. While the female gender was the risk factors associated with EV71 seropositivity in this study, and this conclusion was based the analysis of in preschool children in 2017. The risk factors could be analyzed including other ages.

**Response:** Thank you very much for your suggestion. We analyzed the risk factors in the other age groups, which are shown in the text: For the 6 to 15 year-old children, no significant risk factors were found except older age. For 16-50-year-old people, the significant risk factor associated with EV71 seropositivity was having children in addition to older age: 61% of seropositive 16-50-year-old people had children in their families but only 27% of seronegative 16-50-year-old people had children in their families (p<0.001). Therefore, we did not find gender difference in the other age groups.
